# Supplementary material for: Causes and trends of under-five mortality in the Gilgel Gibe Health and Demographic Surveillance System, 2005 to 2019, Southwest Ethiopia: Cohort study
Source: PLoS One. 2026 Jun 1;21(6):e0349572. doi: 10.1371/journal.pone.0349572 (PMC13225375; doi:10.1371/journal.pone.0349572)
Supplement: Attachment — Submitted filename: Response to reviewers.docx [file pone.0349572.s002.docx]

**Point by point response to the comments**

Dear Editor,

Thank you for the comments and recommendations provided on our manuscript titled “Causes and Trends of Under-five Mortality in Gilgel Gibe Health and Demographic Surveillance System, Southwest Ethiopia.” We have addressed your comments, as well as those from the reviewers, and provide our detailed responses below.

**Journal Requirements:**

If you are reporting a retrospective study of medical records or archived samples, please ensure that you have discussed whether all data were fully anonymized before you accessed them and/or whether the IRB or ethics committee waived the requirement for informed consent. If patients provided informed written consent to have data from their medical records used in research, please include this information

**Response:** The required information is already included in the Ethical Considerations section of the Methods, as well as in the submission form.

3. a) If there are ethical or legal restrictions on sharing a de-identified data set, please explain them in detail (e.g., data contain potentially identifying or sensitive patient information, data are owned by a third-party organization, etc.) and who has imposed them (e.g., a Research Ethics Committee or Institutional Review Board, etc.). Please also provide contact information for a data access committee, ethics committee, or other institutional body to which data requests may be sent.

**Response:** The data used in this study are held by the Jimma University Health and Demographic Surveillance System (HDSS) Coordination Office. Data access is regulated by Jimma University and permitted only through a formal data-sharing agreement. Researchers who wish to access the data may submit a formal request to the Jimma University HDSS Coordination Office, which oversees data governance. **Data Access Contact:** Jimma University Health and Demographic Surveillance System Coordination Office

4. We note that Figure 1 in your submission contain map images which may be copyrighted. All PLOS content is published under the Creative Commons Attribution License (CC BY 4.0), which means that the manuscript, images, and Supporting Information files will be freely available online, and any third party is permitted to access, download, copy, distribute, and use these materials in any way, even commercially, with proper attribution. For these reasons, we cannot publish previously copyrighted maps or satellite images created using proprietary data, such as Google software (Google Maps, Street View, and Earth). For more information, see our copyright guidelines: <http://journals.plos.org/plosone/s/licenses-and-copyright>.

We require you to either present written permission from the copyright holder to publish these figures specifically under the CC BY 4.0 license, or remove the figures from your submission:

**Response:** We obtained a formal permission letter from the Jimma University Health and Demographic Surveillance System. The letter has been included in the online submission as part of the required supporting documents

**Additional Editor Comments:**

Please address the reviewer comments and revise your references to include the most recent sources where possible. Additionally, kindly correct any typographical and grammatical errors throughout the document.

 Response: Dear Editor,

Thank you for your valuable comments. We have revised the references as requested. The following references have been replaced:

- **Reference 27**
  Weldearegawi B, Melaku YA, Abera SF, Ashebir Y, Haile F, Mulugeta A, et al. Infant mortality and causes of infant deaths in rural Ethiopia: A population-based cohort of 3684 births. BMC Public Health. 2015;15(1):1–7. has been replaced by: Ahmed AT, Farah AE, Ali HN, et al. Determinants of early neonatal mortality: a hospital-based retrospective cohort study in the Somali region of Ethiopia. Scientific Reports. 2023;13:1114. <https://doi.org/10.1038/s41598-023-28357-x>
- **Reference 20**
  Nabukalu D, Ntaro M, Seviiri M, Sundararajan R, Reyes R, Boyce R, et al. Using verbal autopsies to estimate under-5 mortality at household level in a rural area of southwestern Uganda: a cross-sectional study. Lancet Global Health. 2018;6:S24.*has been replaced by:* United Nations Inter-agency Group for Child Mortality Estimation (UN IGME). Child Mortality Estimates, 2024 Report.

[Note: HTML markup is below. Please do not edit.]

Reviewers' comments:

5. Review Comments to the Author

Please use the space provided to explain your answers to the questions above. You may also include additional comments for the author, including concerns about dual publication, research ethics, or publication ethics. (Please upload your review as an attachment if it exceeds 20,000 characters)

**Reviewer #1:** Thank you for efforts towards ending under five mortality in Ethiopia.
This manuscript should be editted by professionals to improve its grammar.

**Response:** Thank you for your comments. The manuscript has been edited by a professional editor, and the grammar and language have now been improved
Clearly highlight the type of study this is, in the title whether cohort, case control or crossectional study.

**Response:** thank you for your invaluable suggestion. The title is modified to ‘Causes and Trends of Under-five Mortality: A Retrospective Cohort Study Using Gilgel Gibe Health and Demographic Surveillance System 2005 to 2019, Southwest Ethiopia’.
Also avoid using acronyms before stating them in full as the case in lines 79 and 88

**Response**: Thank you for your comments. All the comments have been thoroughly addressed in the revised manuscript.
Kindly state the bias arising out of the stated limitations

**Response:** thank you very much for your critical comment and it is corrected accordingly. The following statement is included in the manuscript to state a bias that may arise: ‘This may introduce temporal selection bias in which the cause of death identified through VA might not be generalizable to the period where data is missing’

**Reviewer #2:** The title should be adjusted to: Causes and Trends of Under-five Mortality in Gilgel Gibe Health and Demographic
Surveillance System 2005 to 2019, Southwest Ethiopia. The rest of the article is good and scientifically sound.

**Response:** Thank you very much for your insightful comment. The recommendation has been considered and incorporated accordingly.
