# Supplementary material for: Causes and trends of under-five mortality in the Gilgel Gibe Health and Demographic Surveillance System, 2005 to 2019, Southwest Ethiopia: Cohort study
Source: PLoS One. 2026 Jun 1;21(6):e0349572. doi: 10.1371/journal.pone.0349572 (PMC13225375; doi:10.1371/journal.pone.0349572)
Supplement: Attachment — Submitted filename: Response_to_Reviewers_auresp_2.docx [file pone.0349572.s003.docx]

**Point by point response to the comments**

Dear Editor,

Thank you for the comments and suggestions provided on our manuscript titled “Causes and Trends of Under-five Mortality: A Retrospective Cohort Study Using Gilgel Gibe Health and Demographic Surveillance System, 2005 to 2019, Southwest Ethiopia.” We have addressed your comments and suggestions.

The following are the comments and suggestions from the editor along our responses.

1. **Modify the title for clarity and consistency.**

The title currently reads: *"Causes and Trends of Under-five Mortality: A Retrospective Cohort Study Using Gilgel Gibe Health and Demographic Surveillance System, 2005 to 2019, Southwest Ethiopia"*.
I suggest revising it to a more concise and standard format: *"Causes and Trends of Under-five Mortality in the Gilgel Gibe Health and Demographic Surveillance System, Southwest Ethiopia: Cohort Study"*. This places the study design at the end, which aligns with PLOS ONE style.

**Response:** Thank you for your suggestion, and we have modified the title accordingly.

2. **Strengthen the introduction to better articulate the problem, policy efforts, gaps, and study contribution.**

The introduction should be restructured to clearly guide the reader through:

Burden of the problem: Global and national under-five mortality figures, with emphasis on Ethiopia's context and regional disparities.

Causes and consequences: Briefly highlight the leading causes of death and their impact on families, health systems, and national development.

Policy efforts and gaps: Discuss existing national and global strategies (e.g., SDGs, Child Survival Action), and acknowledge that despite these efforts, progress remains uneven. Emphasize the persistent data gaps—particularly the lack of reliable, long-term, community-level data on causes and trends.

What has not been done: Note that most available data come from cross-sectional surveys or facility-based records, which may not capture the true burden in rural communities.

What this study adds: Conclude by clearly stating how this study fills these gaps—by leveraging HDSS data and verbal autopsy to provide robust, community-level evidence on both trends and causes of under-five mortality in Southwest Ethiopia.

**Response:** Thank you very much for your detail comments and suggestions. We have tried to address your comments and incorporated your suggestions.

**3. Clarify the period for verbal autopsy (VA) data.**

In the abstract (lines 52–54) and methods (lines 122–124), the VA period is stated as 2009–2016. However, in the results (lines 206–208) and limitations (lines 361–363), the period is given as 2008–2015. Please verify and unify these dates throughout the manuscript (including tables and figures) to ensure consistency.

**Response**: Thank you for your comments and suggestions. Now it is corrected accordingly, all VA years are 2009 to 2016, including in tables and figures.

**4. Address discrepancies in VA data years in figures and tables.**

Table 1 and Table 2 are labeled as "2009 to 2016," but the results text refers to "2008 to 2015." Please ensure all elements (abstract, methods, results, tables, and figures) reflect the same correct time frame.

**Response:** Thank you for your comments. The dates in the manuscript regarding VA data are corrected to 2009 to 2016 in both texts and tables and graphs.

**5. Provide the specific p-values or confidence intervals for the Mann-Kendall trend test.**
In lines 273–274, you report tau values and two-sided p-values (e.g., p=1.63e−05p=1.63e−05). Please also present these in a more reader-friendly format (e.g., p<0.001p<0.001) and ensure they are clearly labelled in the text or in a supplementary table.

**Response:** Thank you for your comments, and they are corrected accordingly.

**6. Correct minor typographical and grammatical errors.**

- Line 217: "were (Delete were) occurred" – please revise to "occurred".
- Line 269: "alue" should be "tau value".
- Line 276: "Delete the" before "Figure 5" – please correct.
- Line 279: "Delete a" before "smooth" – please correct.
- Line 305: "was (Delate was) occurred" – please revise to "occurred".

**Response:** The comments are addressed accordingly.

**7. Strengthen the interpretation of the LOESS smoothed trend.**

You mention that the LOESS model "revealed a relatively smooth declining pattern" (lines 277–280). Please add a brief statement on what this smoothing adds beyond the raw data (e.g., confirming that the decline is consistent despite year-to-year fluctuations).

**Response:** Thank you for your comments and suggestions. The we addressed the comments and suggestions accordingly.

**8. Strengthen the discussion with clear implications for policy, research, and practice.**

The discussion should be restructured to include a dedicated section or paragraph explicitly addressing implications. Please ensure the following:

**Implications for policy**

Based on the finding recommend strengthening perinatal and neonatal care.

Given that infectious diseases remain leading causes in post-neonatal and child age groups, emphasize the need for integrated interventions: immunization, nutrition programs, water and sanitation, and malaria prevention.

Highlight the finding that over 60% of deceased children did not visit a health facility, suggesting critical barriers to access.

**Implications for research**

Note that VA data were only available for part of the surveillance period. What should you recommend?

Implication for practice????

**Response:** Thank you for the detail comments and suggestions that improve the manuscript. We have tried to address your suggestions and comments.

**9.  Expand on the "undetermined" causes of death.**

In Table 2, a small proportion of deaths are listed as "undetermined" (e.g., 2.8% in neonates, 6.3% in post-neonates). Please comment in the discussion on whether this proportion is consistent with other HDSS studies and whether it could affect the interpretation of cause-specific mortality.

**Response:** Thank you for the comments and we have incorporated the comments.

**10. Address the potential impact of the COVID-19 period.**

Although your study ends in 2019, consider adding a brief note in the discussion that the pre-pandemic trends observed may serve as a baseline for future studies evaluating the impact of COVID-19 on under-five mortality in Ethiopia.

**Response:** Thank you for suggesting us such important point. We have included it accordingly.

**11. Verify the data availability statement.**

You state in lines 375–376: *"The data used in this study is available from the corresponding author on reasonable request."* However, in the submission form, you indicate that data are held by the Jimma University HDSS Coordination Office and require a formal data-sharing agreement. Please align the in-text statement with the journal’s requirement to specify the third-party data access contact. I recommend using the more detailed statement already provided in the submission form.

These revisions will further strengthen the clarity, consistency, and overall scientific rigor of your manuscript. We look forward to seeing your revised version.

**Response**: Thank you for your suggestion. We have updated the data availability statement based on your suggestion.
